# The cost of perspective switching: Constraints on simultaneous activation

**DOI:** 10.3758/s13423-024-02633-x

**Published:** 2025-01-13

**Authors:** Dorit Segal

**Affiliations:** Department of Education and Psychology, The Open University, 1 University Road, P.O. Box 808, 4353701 Ra’anana, Israel

**Keywords:** Switching, Mixing, Mental rotation, Vincentile analysis

## Abstract

Visual perspective taking often involves transitioning between perspectives, yet the cognitive mechanisms underlying this process remain unclear. The current study draws on insights from task- and language-switching research to address this gap. In Experiment 1, 79 participants judged the perspective of an avatar positioned in various locations, observing either the rectangular or the square side of a rectangular cube hanging from the ceiling. The avatar's perspective was either consistent or inconsistent with the participant’s, and its computation sometimes required mental transformation. The task included both single-position blocks, in which the avatar's location remained fixed across all trials, and mixed-position blocks, in which the avatar's position changed across trials. Performance was compared across trial types and positions. In Experiment 2, 126 participants completed a similar task administered online, with more trials, and performance was compared at various points within the response time distribution (vincentile analysis). Results revealed a robust switching cost. However, mixing costs, which reflect the ability to maintain multiple task sets active in working memory, were absent, even in slower response times. Additionally, responses to the avatar's position varied as a function of consistency with the participants' viewpoint and the angular disparity between them. These findings suggest that perspective switching is costly, people cannot activate multiple perspectives simultaneously, and the computation of other people's visual perspectives varies with cognitive demands.

## Introduction

Visual perspective taking (VPT) is vital for communication, as it enables us to predict whether other people can see what we see (Level-1 VPT), and how they view objects or scenes (Level-2 VPT; Flavell et al., 1981). Research on VPT has focused on how different perspectives (“self” or “other”), or the consistency between them, affect response time and accuracy. Samson et al. (2010) used the dot task (Level-1 VPT) to examine how participants verify the number of dots in a three-dimensional (3D) room, either from their own viewpoint or from the viewpoint of an on-screen avatar. Many researchers assume that performance on such tasks involves automatic computation of both perspectives, and that interference occurs when perspectives are inconsistent (Samson et al., 2010; Qureshi et al., 2010; Qureshi & Monk, 2018). Studying Level-2 VPT, Michelon and Zacks (2006) manipulated the angular disparity between the participant and the avatar (0º, 90º, 180º, 270º), and showed that increased distance led to slower responses, suggesting that participants mentally transform their position to align it with the avatar's position (Surtees et al., 2013; Thirioux et al., 2010). Transformation occurs predominantly when orientation disparity exceeds 80° (Kessler & Rutherford, 2010; Kessler & Thomson, 2010).

These studies overlook a critical factor in VPT, as the transition between perspectives from trial to trial may also affect performance. In Samson et al.'s (2010) task, continuous judgment of self-perspective led to faster responses relative to switching between perspectives. Ferguson et al. (2017) tracked participants' eye movements on the dot task. In half the trials, consecutive trials presented the same perspective (stay trials), and in the other half, the perspectives switched (switch trials). Participants responded more quickly on stay than on switch trials, indicating a switching cost.

Martin et al. (2019) presented street scenes to 122 younger (M_age_ = 24 years) and 50 older adults (M_age_ = 66 years). Participants assessed the number of balls that they or an avatar saw (Level-1 VPT), and determined whether there were more balls on the avatar's left or right side (Level-2 VPT). The results revealed a switching cost from self-perspective to the other's perspective on both VPT levels, especially in older adults. Yuan et al. (2023) explored Level-2 VPT in 31 children (M_age_ = 10.5 years) and 41 adults (M_age_ = 20.5 years). Participants judged numerals from their own or from an avatar's perspective. Following inconsistent trials in which participants judged the avatars' perspective, they were slower to judge their own perspective. The authors explained that inhibition of self-perspective makes it harder to reactivate this perspective on the following trial. These studies suggest that switching between perspectives incurs a cost, and this cost may affect the processing of subsequent trials.

Task-switching studies have consistently demonstrated that switching between tasks takes longer than repeating a task (e.g., Huff et al., 2015; Meuter & Allport, 1999; Prior & Gollan, 2011). This cost reflects carry-over effects from the previous task as well as the time needed for reconfiguration (Monsell, 2003; Rogers & Monsell, 1995). However, the mechanisms involved in visual perspective switching may differ from other types of switching, as prior research suggests that switching costs may entail distinct processes across different tasks and may even vary within the same task across trials (Huff et al., 2015; Segal et al., 2019).

Huff et al. (2015) asked 570 adults (aged 18–94 years) to classify whether the letter in a letter-number stimulus was a consonant or a vowel, and whether the number was odd or even. Participants completed single-task blocks (i.e., classifying only the letter) or mixed-tasks blocks, which consisted of similar (“stay” trials) or different (“switch” trials) classifications. Responses were faster on stay than on switch trials, demonstrating a switching cost, and faster on single-task trials than on stay trials, showing a mixing cost. This mixing cost is thought to reflect the ability to maintain multiple task sets active in working memory and focus on the relevant one (Huff et al., 2015; Marí-Beffa & Kirkham, 2014). To assess control over switching, the authors ranked latencies for single, stay, and switch trials within each participant from fastest to slowest, and grouped the data into time bins (vincentile analysis). This analysis showed that switching costs were less noticeable in slow responses, presumably because these responses reflect task uncertainty, ineffective memory retrieval (Balota et al., 2008; Schmiedek et al. 2007), or lapses of attention (West, 1996; West et al., 2002). According to Huff et al. (2015), task uncertainty led participants to activate both the target and the competitor on stay trials, slowing responses on stay trials, as the competitor interfered with target selection, and reducing the ability to focus on the relevant task (Marí-Beffa & Kirkham, 2014). Co-activation also reduced switching costs, because the competitor was available prior to switch trials. Additionally, the activation of the competitor on stay trials amplified the difference in latencies between single and stay trials, thus increasing mixing costs.

Huff et al.'s (2015) findings suggest that the ability to activate multiple task-sets simultaneously affects both switching and mixing costs, particularly when responses are slow and when participants can prepare for subsequent responses (Koch, 2005). Segal et al. (2019) used a similar analysis to examine how 98 bilinguals switched between judging colors and shapes of objects and how they shifted between languages when naming digits. The findings revealed smaller switching costs for slow responses only in the color-shape task. The authors explained that colors and shapes, much like letters and numbers, naturally co-occur (e.g., a yield sign is a red triangle). Thus, co-activation is the default under task uncertainty. Conversely, language production requires the selection of one language, even under task uncertainty, because participants cannot say a word in two languages together. Selecting a single language on stay trials makes switching more difficult. Hence, the nature of representations and the degree to which they co-occur affect switching mechanisms.

According to some theories, when people adopt another person's perspective, they generate both self and other viewpoints and then select the relevant one, especially in Level-1 VPT (e.g., Leslie & Thaiss, 1992; Leslie et al., 2005; Ramsey et al., 2013). These theories imply that people can activate various perspectives before inhibiting the unintended one (Nilsen & Graham, 2009; Qureshi et al., 2010; Yuan et al., 2023). However, Level-2 VPT tasks may require mental transformation, making simultaneous activation implausible, and introducing the need for a switching component.

The current study aims to investigate whether participants can activate various visual perspectives simultaneously or must switch between them, and whether perspective-taking varies with task demands.

## Study 1

### Method

#### Transparency and openness

Reference to sample size decisions and all data exclusions and manipulations appear below. All statistical analyses used SPSS 27, except for Bayesian analyses that used JASP (Van Doorn et al., 2021). The data as well as the analytic code are available at: https://osf.io/usfxe/. The study was not pre-registered.

#### Participants

Eighty participants were recruited for the study, but one was excluded for not meeting the inclusion criteria. Thus, 79 participants (63% female), aged 18–40 years (*M* = 26.03, *SD* = 5.00), took part in the study at The Open University in exchange for course credit. Years of education ranged between 12 and 23 years (*M* = 14.04, *SD* = 2.00). Exclusion criteria included self-reported diagnosis of reading disorders, attention deficit disorder (ADHD), and uncorrected visual problems. The G*Power3 program (Faul et al., 2007) served to estimate the necessary sample size for conducting an analysis of variance (ANOVA) involving two within-subject variables. Based on a significance level of .05 and a statistical power of .85, a small effect size of *f* = .033, along with a conservative estimate for the correlation among repeated measures (*r* = .5), the software computed a recommended sample size of 32 participants. However, to counterbalance research conditions across participants, and include enough participants in each order of presentation, the sample size was larger.

### Tools

#### Visual perspective switching task

**Fig. 1:**
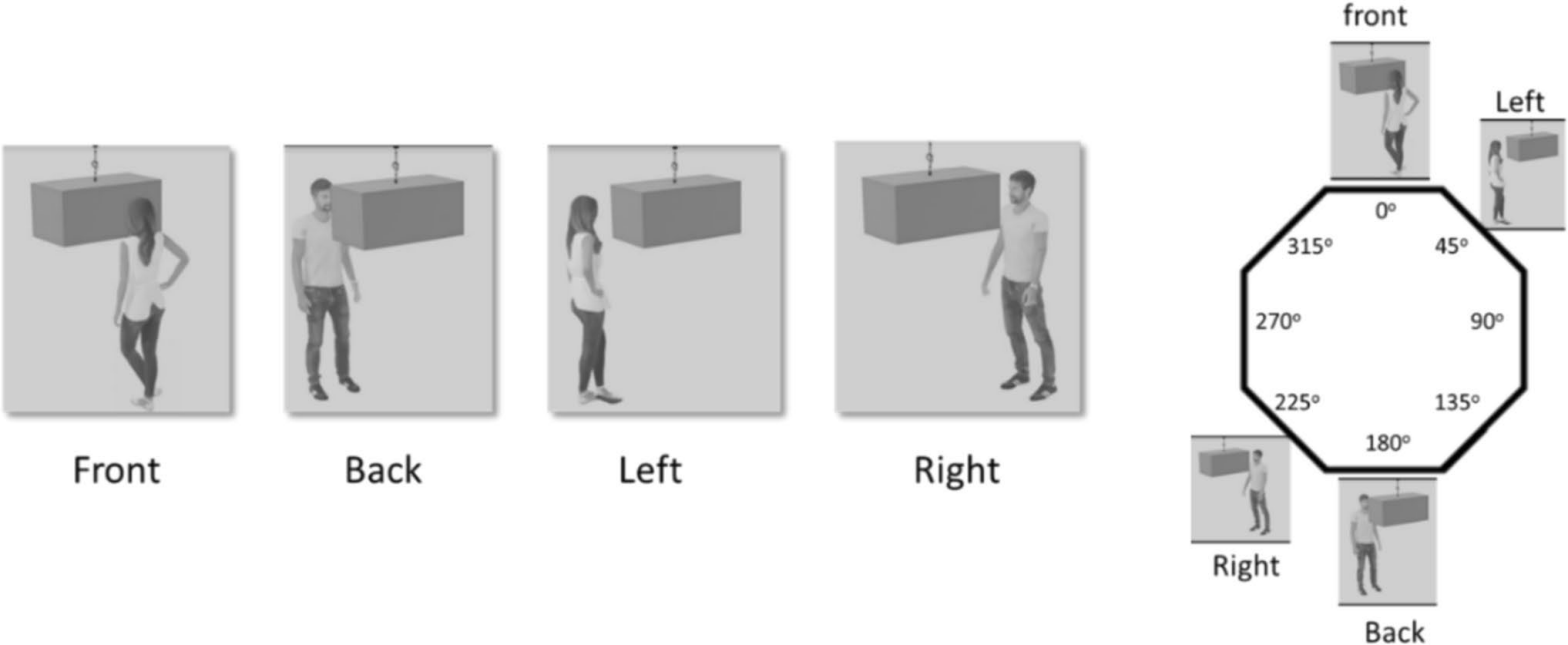
Avatar's position by orientation disparity

Blender 4.0 (Blender Foundation, Amsterdam, the Netherlands) served to create 16 3D scenes. Each scene featured an avatar (50% female) who faced a rectangular cube hanging from the ceiling (see Fig. 1). The avatar's placement relative to the cube created different Level-2 VPT scenarios. As shown on Fig. 1, the avatar stood either in front of the cube (≈ 0° orientation disparity, front), behind it (≈ 180° disparity, back), to its left (≈ 45° disparity, left), or to its right (≈ 225° disparity, right). Note that the cube is tilted, and the avatar has been positioned at an approximate angle to ensure that the participant can clearly understand (or imagine) what the avatar sees. Each position occurred on 25% of the trials, with positions varying in consistency with the participant's perspective and with the need for mental transformation, leading to different cognitive demands (see Table 1).

**Table 1:** Characteristics of avatar's position, by consistency and mental transformation requirements

Position	Consistent with the participant's perspective	Requires egocentric transformation
Front	**+**	**-**
Back	**+**	**+**
Left	**-**	**-**
Right	**-**	**+**

**Fig. 2:**
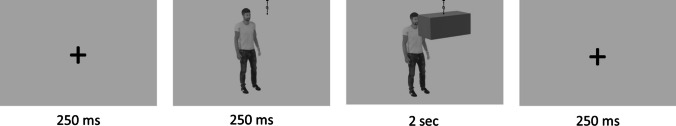
The experimental design

Each trial began with a fixation cross that remained on the screen for 250 ms. Then the avatar appeared for 250 ms (as in Segal et al., 2019), followed by the appearance of the cube (see Fig. 2). The avatar and the cube remained on the screen until response, or for a maximum of 2,000 ms. Participants pressed the "L" key if the avatar saw a square and the "A" key if the avatar saw a rectangle. Response times were measured from the appearance of the cube until the participant responded.

**Fig. 3:**
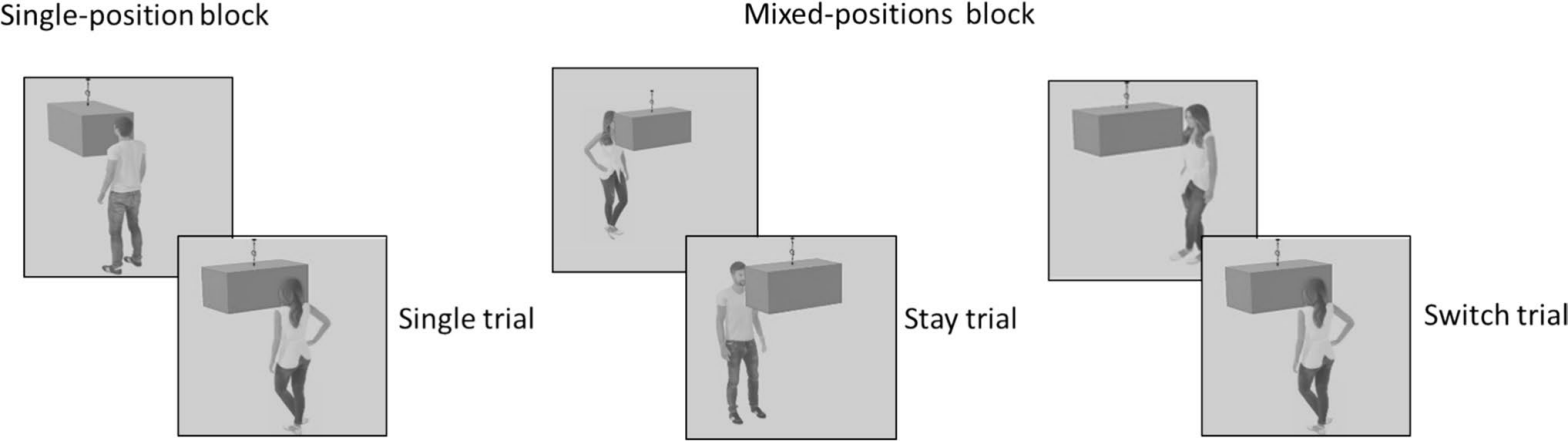
Examples of single, stay, and switch trials, by block type

Participants first completed four single-position blocks (see Fig. 3). Each block began with eight practice trials followed by a dummy trial (a warm-up trial similar to target trials) and by eight target trials. In all trials within each single-position block, the avatar stood at a fixed position, at the front, back, left, or right side of the cube. To prevent strategic responses and reduce early response preparation, the orientation of the cube varied across trials: on half the trials its square side faced forward, and on the other half its rectangular side faced forward. Half the trials required the same response as the previous trial, and the other half required a change in response. Importantly, consecutive trials never presented the exact same image. The single position blocks were counterbalanced across participants, with 25% of the participants starting in each position (front, back, left, or right). After completing these blocks, participants performed eight mixed-position practice trials followed by four mixed-position blocks, each containing a dummy trial and 16 additional target trials. In these blocks, the avatar's position switched across trials in a pseudo-random order. Half the trials maintained the same position on consecutive trials (“stay” trials) and in the other half the positions of the avatars switched (“switch” trials, see Fig. 3). Consecutive trials never presented the exact same image. Lastly, participants completed additional four single-position blocks. For each participant, the order of these blocks was different from the order of the initial single-position block to avoid order effects.

#### Procedure

Participants signed an informed consent and provided demographic information online through the Qualtrics platform. Then they performed the Visual Perspective Switching task through PsychoPy 2001.1.4 (Peirce, 2009). Participants completed the task on a desktop computer at the university lab in a single 30-min session.

#### Data analysis

Two-way repeated-measures analyses of variance (ANOVA) were used to determine whether perspective switching and perspective mixing incur a cost. Each ANOVA examined the effects of trial type (stay and switch; single and stay) and position (front, back, left, and right) on VPT, either for latency or for accuracy.

### Results and discussion

Dummy trials, incorrect responses (4%), responses following an error (4%), and responses either faster than 150 ms (<1%) or slower than 3 SDs from the participant's mean reaction time (RT) (3%) were excluded. Table 2 shows mean RTs and accuracy rates by trial types and positions.

**Table 2:** Mean (and SE) reaction times (RTs; ms) and accuracy rates (%) by trial types and positions

		Front	Back	Left	Right	Overall
RT	Single	516.78 (8.10)	529.70 (8.72)	538.35 (8.30)	571.32 (9.80)	539.03 (7.77)
	Stay	517.38 (7.77)	542.38 (8.82)	554.74 (10.36)	566.32 (9.97)	545.20 (8.39)
	Switch	542.56 (8.79)	570.73 (9.60)	592.93 (10.17)	585.55 (10.25)	572.94 (8.90)
	Overall	525.57 (7.51)	547.60 (8.13)	562.01 (8.94)	574.40 (9.32)	
Accuracy	Single	.98 (.01)	.96 (.01)	.94 (.01)	.93 (.01)	.95 (.01)
	Stay	.97 (.01)	.98 (.01)	.97 (.01)	.96 (.01)	.97 (.01)
	Switch	.97 (.01)	.97 (.01)	.94 (.01)	.94 (.01)	.96 (.01)
	Overall	.97 (.00)	.97 (.01)	.95 (.01)	.94 (.01)	

#### Reaction time (RT) analysis

##### Switching cost

A repeated-measures ANOVA that compared stay and switch trials showed a significant difference*, F* (1, 78) = 75.13, *MSE* = 1618, *p* < .001, *n*^2^ = .491, with faster responses on stay than on switch trials. The main effect of position was also significant, *F* (3, 234) = 35.58, *MSE* = 2007, *p* < .001, *n*^2^ = .313. Paired-samples t-tests demonstrated the fastest responses in the front position, followed by the back, left, and right positions (*p* < .002 for all paired comparisons). However, RTs for the left and the right positions were similar (*p* = .649). The interaction between trial type and position was not significant, *F* (3, 234) = 1.95, *MSE* = 1274, *p* = .122, *n*^2^ = .024.

##### Mixing cost

A repeated-measures ANOVA that compared single and stay trials showed a non-significant difference*, F* (1, 78) = 3.32, *MSE* = 1811, *p* = .072, *n*^2^ = .041. The main effect of position was significant, *F* (3, 234) = 39.04, *MSE* = 1884, *p* < .001, *n*^2^ = .334. Paired-samples t-tests demonstrated fastest responses in the front position, followed by the back, left, and right positions (*p* < .036 for all paired comparisons). The interaction between trial type and position was significant, *F* (3, 234) = 2.66, *MSE* = 1496, *p* = .049, *n*^2^ = .033. A Bayesian ANOVA revealed moderate evidence against including trial type as a factor (BF_10_ = 0.397), suggesting that the null effect regarding mixing costs reflects a true lack of a difference between single and stay trials.

#### Accuracy analysis

##### Switching cost

A repeated-measures ANOVA that compared stay and switch trials showed a significant difference*, F* (1, 78) = 4.14, *MSE* = .004, *p* = .045, *n*^2^ = .050, with greater accuracy on stay than on switch trials. The main effect of position was also significant, *F* (3, 234) = 3.32, *MSE* = .007, *p* = .021, *n*^2^ = .041. Paired-samples t-tests found greater accuracy on front and back positions than on right position (both* p* < .034), but all other comparisons did not reach significance (all *p*s > .055). The interaction between trial type and position was not significant, *F* (3, 234) = 1.20, *MSE* = .004, *p* = .311, *n*^2^ = .015.

##### Mixing cost

A repeated-measures ANOVA that compared single and stay trials showed a significant difference*, F* (1, 78) = 6.30, *MSE* = .005, *p* = .014, *n*^2^ = .075. Participants responded more accurately on stay than on single trials. The main effect of position was also significant, *F* (3, 234) = 4.09, *MSE* = .008, *p* = .007, *n*^2^ = .050. Paired-samples t-tests found greater accuracy on the front position than on either the left (*p* = .043) or the right positions (*p* = .007), and participants were more accurate on the back position than on the right position (*p* = .019). No other comparison reached significance (all *p*s > .135). The interaction between trial type and position was not significant, *F* (3, 234) = 2.21, *MSE* = .005, *p* = .087, *n*^2^ = .028.

These findings support the assumption that perspective switching incurs a cost, and that it is impossible to keep multiple visual perspectives active at the same time.

## Study 2

Study 2 aimed to replicate the findings of Study 1, and to examine whether individuals can activate several perspectives under task uncertainty.

### Methods

#### Transparency and openness

As in Study 1, all statistical analyses used SPSS 27, except for Bayesian analyses that used JASP (Van Doorn et al., 2021). The data as well as the analytic code are available at: https://osf.io/usfxe/. The study was not pre-registered.

#### Participants

One hundred and thirty-four participants were recruited for the study through a survey company. Two of them were excluded due to a technical error and another six were excluded due to extremely low accuracy rates (more than 3 SDs from the group's mean). The final sample included 126 participants (54% female), aged 18–40 years (*M* = 30.07, *SD* = 5.24), with 12–24 years of education (*M* = 14.96, *SD* = 2.16).

#### Tools and procedure

As in Study 1, participants completed a demographic questionnaire and then an online Visual Perspective Switching Task. The task was similar to that in Study 1, but the number of trials in the mixed blocks was doubled, ensuring an equal number of single, stay, and switch trials (64 trials per condition) and providing sufficient trials for examining switching and mixing costs across the RT distribution. Additionally, the instructions were supplemented with examples to clarify the correct responses, and participants received auditory feedback for incorrect responses during the practice blocks. The task was administered using PsychoPy version 2001.1.4 (Peirce, 2009) through the Pavlovia website. PsychoPy has been demonstrated to be one of the most precise and accurate software options for measuring reaction times in both in-lab and online settings (Bridges et al., 2020).

#### Data analysis

The analyses were similar to those conducted in Study 1. In addition, a vincentile analysis was conducted to investigate the possibility of simultaneous co-activation of visual perspectives, focusing on switching and mixing costs. In this analysis, the RTs of each participant on every trial type were ranked from fastest to slowest, independent of position, following methods outlined in Huff et al. (2015) and Segal et al. (2019). The ranked RTs were then divided into five time bins. The fastest 20% of RTs were averaged into bin 1, the subsequent 20% into bin 2, and so forth. The slowest 20% of trials were averaged into bin 5. Each bin contained a roughly equal number of trials, although slight discrepancies occurred due to varying numbers of error responses. Finally, to investigate switching and mixing costs across the entire RT distribution, two additional repeated-measures ANOVAs were conducted with trial type (stay vs. switch in the first ANOVA; single vs. stay in the second ANOVA) and bin number (1–5) as within-subject variables.

### Results and discussion

Dummy trials, incorrect responses (6%), responses following an error (6%), and responses either faster than 150 ms (<1%) or slower than 3 SDs from the participant's mean RT (1%) were excluded. Table 3 shows mean RTs and accuracy rates by trial types and positions.

**Table 3:** Mean (and SE) reaction times (RTs; ms) and accuracy rates (%) by trial types and positions

		Front	Back	Left	Right	Overall
RT	Single	580.34 (14.42)	587.10 (14.56)	593.34 (15.19)	628.22 (14.66)	597.25 (14.68)
	Stay	578.85 (13.84)	607.14 (13.95)	607.31 (14.76)	625.07 (14.12)	604.59 (14.17)
	Switch	595.20 (13.66)	643.79 (14.05)	641.50 (15.29)	641.82 (14.62)	630.58 (14.39)
	Overall	584.78 (13.95)	612.68 (14.19)	614.05 (15.06)	631.70 (14.45)	
Accuracy	Single	.95 (.01)	.95 (.01)	.93 (.01)	.93 (.01)	.94 (.01)
	Stay	.96 (.01)	.96 (.01)	.93 (.01)	.93 (.01)	.95 (.01)
	Switch	.96 (.01)	.96 (.01)	.93 (.01)	.92 (.01)	.94 (.01)
	Overall	.96 (.01)	.96 (.01)	.93 (.01)	.93 (.01)	

#### RT analysis

##### Switching cost

A repeated-measures ANOVA that compared stay and switch trials showed a significant difference*, F* (1, 124) = 76.42, *MSE* = 2277, *p* < .001, *n*^2^ = .381, with faster responses on stay than on switch trials. The main effect of position was also significant, *F* (3, 372) = 58.62, *MSE* = 1979, *p* < .001, *n*^2^ = .321. Paired-samples t-tests demonstrated faster responses in the front position than in the back, left, and right positions (*p* < .001 for all paired comparisons). Responses were faster in the back than in the right (*p* = .018) position, and faster in the left than in the right position (*p* = .028). Responses to back and left positions were similar (*p* = .991). The interaction between trial type and position was significant as well, *F* (3, 372) = 7.28, *MSE* = 1056, *p* < .001, *n*^2^ = .055.

##### Mixing cost

A repeated-measures ANOVA that compared single and stay trials showed a non-significant difference between the two trial types*, F* (1, 124) = 2.18 *MSE* = 2823, *p* = .142, *n*^2^ = .017. The main effect of position was significant, *F* (3, 372) = 44.93, *MSE* = 2046, *p* < .001, *n*^2^ = .266. Paired-samples t-tests demonstrated faster responses in the front position than in the back, left, and right positions (*p* < .001 for all paired comparisons), with no difference between the back and the left positions (*p* = .475). The slowest responses were in the right position (*p* < .001 for all paired comparisons). The interaction between trial type and position was significant, *F* (3, 372) = 4.93, *MSE* = 1603, *p* = .002, *n*^2^ = .038. A Bayesian ANOVA confirmed that the null effect in mixing costs reflected a true lack of difference between single and stay trials (F_10_ = 0.249).

#### Accuracy analysis

##### Switching cost

A repeated-measures ANOVA that compared stay and switch trials showed a significant difference*, F* (1,125) = 4.49, *MSE* = .003, *p* = .036, *n*^2^ = .035, with greater accuracy on stay than on switch trials. The main effect of position was also significant, *F* (3, 375) = 11.21, *MSE* = .008, *p* < .001, *n*^2^ = .082. Paired-samples t-test comparisons showed similar accuracy rates for front and back positions (*p* = .894), and responses on these two positions were more accurate than responses to left and right positions (all *p*s < .002). Accuracy was similar for the left and right positions (*p* = .345). The interaction between trial type and position was not significant, *F* (3, 375) = 1.67, *MSE* = .003, *p* = .173, *n*^2^ = .013.

##### Mixing cost

A repeated-measures ANOVA showed similar accuracy rates for single and stay trials*, F* (1, 125) = 1.08, *MSE* = .013, *p* = .301, *n*^2^ = .009. The main effect of position was significant, *F* (3, 375) = 5.99, *MSE* = .008, *p* < .001, *n*^2^ = .046. Paired-samples t-test comparisons showed similar accuracy rates for front and back positions (*p* = .737), and responses on these two positions were more accurate than responses on the left and right positions (all *p*s < .010). Accuracy was similar for the left and right positions (*p* = .771). The interaction between trial type and position was not significant, *F* (3, 375) = .158, *MSE* = .004, *p* = .925, *n*^2^ = .001. A Bayesian ANOVA confirmed that the null effect in mixing costs reflected a true lack of difference between single and stay trials (F_10_ = 0.208).

#### Vincentile analysis

**Fig. 4:**
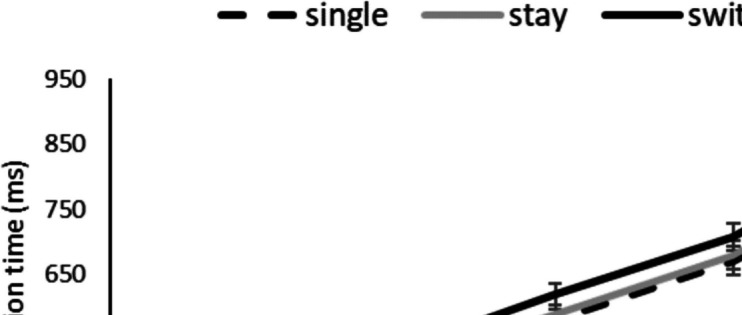
Mean (and SE) reaction time across vincentile bins, by trial type

Figure 4 illustrates the mean RTs across bins and trial types.

##### Switching cost

A repeated-measures ANOVA with trial type (stay and switch) and bin number (1–5) revealed a significant effect of trial type, *F* (1, 125) = 71.21, *MSE* = 3054, *p* < .001, *n*^2^ = .363, as well as a significant effect of bin number, *F* (4, 500) = 222.65, *MSE* = 27,378, *p* < .001, *n*^2^ = .640. The interaction between bin number and trial type was not significant, *F* (4, 500) = 1.36, *MSE* = 627, *p* = .245, *n*^2^ = .011.

##### Mixing cost

A repeated-measures ANOVA with trial type (single and stay) and bin number revealed no significant effect of trial type, *F* (1, 125) = 3.270, *MSE* = 5496, *p* = .073, *n*^2^ = .025. The effect of bin number was significant, *F* (4, 500) = 210.88, *MSE* = 28,007, *p* < .001, *n*^2^ = .628, but the interaction of trial type and bin number was not significant, *F* (4, 500) = .350, *MSE* = 950, *p* = .844, *n*^2^ = .003.

These findings show that participants cannot simultaneously activate multiple perspectives under task uncertainty.

## General discussion

Using a novel Level-2 visual perspective-switching task, the current study found a robust switching cost. However, there were no mixing costs, even in the slowest responses that reflect task uncertainty. These findings imply that participants cannot co-activate visual perspectives, and must consider only one perspective at any given time. Thus, any theoretical account of VPT should refer to a switching component.

Participants responded more slowly and less accurately when required to switch between visual perspectives than when maintaining the same perspective across trials. This switching cost was robust, and it appeared irrespective of the avatar's position. The findings align with previous reports of a switching cost in VPT tasks (Ferguson et al., 2017; Martin et al., 2019). The switching cost may reflect carry-over of the previous task set (i.e., the avatar's position in the previous trial), or the time needed for its reconfiguration (Monsell, 2003; Rogers & Monsell, 1995). However, the interaction of the cost with the avatar's position in Study 2 suggests that it may also reflect the time needed for computation of the avatar's perspective, depending on its position.

Participants responded most rapidly and most accurately when the avatar was in front position and its viewpoint aligned with their own. Responses were slower when the avatar stood behind the cube, facing the participant. These results align well with previous findings of faster responses to avatars that shared participants' position than to avatars that faced participants (Michelon & Zacks, 2006). Thus, the results show that participants transform their position to match the avatar's position (Surtees et al., 2013; Thirioux et al., 2010). Taking the left-positioned avatar's perspective required no mental transformation, since it shared the participant's quadrant (Erle & Topolinski, 2017; Kessler & Rutherford, 2010; Kessler & Thomson, 2010), and since participants could observe the avatar's viewpoint. However, RTs increased compared to front and back positions, because the avatar's perspective differed from the participant's perspective, and this inconsistency required cognitive resources. Responses to right-positioned avatars were slowest and least accurate, due to the large angular disparity between the participant and the avatar that required mental transformation, and because of the inconsistency between viewpoints.

Unlike these robust switching costs, the study showed no mixing costs. Participants responded equally fast on single and stay trials although they were less accurate on single trials. However, this accuracy difference was only evident in Study 1, raising questions about its validity. Mixing costs reflect the ability to maintain multiple task-sets accessible, focusing on the relevant task and ignoring the irrelevant one (e.g., Marí-Beffa & Kirkham, 2014; Minear & Shah, 2008; Pettigrew & Martin, 2016). The absence of such costs suggests that participants cannot keep multiple perspectives active simultaneously, and must consider only one perspective at a time. The vincentile analysis further supports these conclusions. Even within a mixed block, in which dual activation could be advantageous, and even under task uncertainty, participants selected only one perspective on stay trials, likely the most recent one. This tendency led to stable switching and mixing costs across the RT distribution. Note that Segal et al. (2019) found an increase in switching and in mixing costs across the RT distribution in the language switching task, probably due to dual activation of both languages prior to production (see also Blumenfeld & Marian, 2013; Macizo, 2016). In contrast, VPT may not allow such co-activation.

Perspective switching can explain why Yuan et al. (2023) found slower responses to self-perspective after judging the avatar's viewpoint. If participants transformed their position to adopt the avatar's position, they had to switch back to their position to make self-judgment. The current findings may also explain why previous studies found an association between switching abilities and perspective-taking in various populations (Bock et al., 2015; Champagne-Lavau et al., 2012; Long et al., 2018; Wang et al., 2015). Future research should investigate whether a switching mechanism is integral also to Level-1 VPT, as well as other perspective-taking tasks.

The study has some limitations. First, the distribution of the avatars' positions across trials was equal, but switching to these positions began in various locations. Future research should examine whether switching costs depend on the starting point. Second, the avatar may have acted as a directional cue, helping participants adopt its visual perspective. However, previous research demonstrated that individuals compute the avatar's perspective automatically (Samson et al., 2010), making this possibility unlikely.

In conclusion, the current study highlights the dynamics of VPT by showing that shifting between perspectives incurs a cost. It also suggests that individuals cannot access multiple perspectives simultaneously, hence emphasizing the essential role of switching in VPT. Consequently, perspective-taking models that aim to explain how individuals adopt other people's perspectives must include a switching mechanism.

## Data Availability

The code used to analyze the data generated in the study is available at: https://osf.io/usfxe/.
